# Comparison of incidence and cost of influenza between healthy and high-risk children <60 months old in Thailand, 2011-2015

**DOI:** 10.1371/journal.pone.0197207

**Published:** 2018-05-17

**Authors:** Wanitchaya Kittikraisak, Piyarat Suntarattiwong, Wiboon Kanjanapattanakul, Darunee Ditsungnoen, Chonticha Klungthong, Kim A. Lindblade, Stefan Fernandez, Fatimah S. Dawood, Tawee Chotpitayasunondh, Sonja J. Olsen

**Affiliations:** 1 Influenza Program, Thailand Ministry of Public Health–U.S. Centers for Disease Control and Prevention Collaboration, Nonthaburi, Thailand; 2 Queen Sirikit National Institute of Child Health, Ministry of Public Health, Bangkok, Thailand; 3 Armed Forces Research Institute of Medical Sciences, Bangkok, Thailand; 4 Influenza Division, U.S. Centers for Disease Control and Prevention, Atlanta, Georgia, United States of America; Public Health Agency of Canada, CANADA

## Abstract

**Introduction:**

Thailand recommends influenza vaccination for children aged 6 months to <36 months, but investment in vaccine purchase is limited. To inform policy decision with respect to influenza disease burden and associated cost in young children and to support the continued inclusion of children as the recommended group for influenza vaccination, we conducted a prospective cohort study of children in Bangkok hospital to estimate and compare influenza incidence and cost between healthy and high-risk children.

**Methods:**

Caregivers of healthy children and children with medical conditions (‘high-risk’) aged <36 months were called weekly for two years to identify acute respiratory illness (ARI) episodes and collect illness-associated costs. Children with ARI were tested for influenza viruses by polymerase chain reaction. Illnesses were categorized as mild or severe depending on whether children were hospitalized. Population-averaged Poisson models were used to compare influenza incidence by risk group. Quantile regression was used to examine differences in the median illness expenses.

**Results:**

During August 2011-September 2015, 659 healthy and 490 high-risk children were enrolled; median age was 10 months. Incidence of mild influenza-associated ARI was higher among healthy than high-risk children (incidence rate ratio [IRR]: 1.67; 95% confidence interval [CI]: 1.13–2.48). Incidence of severe influenza-associated ARI did not differ (IRR: 0.40; 95% CI: 0.11–1.38). The median cost per mild influenza-associated ARI episode was $22 among healthy and $25 among high-risk children (3–4% of monthly household income; difference in medians: -$1; 95% CI for difference in medians: -$9 to $6). The median cost per severe influenza-associated ARI episode was $232 among healthy and $318 among high-risk children (26–40% and 36–54% of monthly household income, respectively; difference in medians: 110; 95% CI for difference in medians: -$352 to $571).

**Conclusions:**

Compared to high-risk children, healthy children had higher incidence of mild influenza-associated ARI but not severe influenza-associated ARI. Costs of severe influenza-associated ARI were substantial. These findings support the benefit of annual influenza vaccination in reducing the burden of influenza and associated cost in young children.

## Introduction

Influenza virus infections account for 3–5 million severe illnesses and an average of 250,000–500,000 deaths globally each year.[1] Influenza vaccination remains the most effective method of influenza prevention. Although influenza affects persons of all ages, young children, older adults, persons with chronic underlying medical conditions, and pregnant women have been identified as groups at increased risk of severe influenza.[2, 3] Studies have documented that children aged <60 months have higher rates of influenza-associated hospitalization compared to older children.[4, 5] Additionally, compared to healthy children, children with underlying cardiac, pulmonary, and neurologic conditions are at even greater risk for influenza-associated hospitalization and severe outcomes.[4, 6–9] However, although children with underlying conditions are known to be at increased risk of severe influenza if infected, it is not known whether they are at increased risk for influenza virus acquisition. There are few data on differences in influenza-associated costs between children with underlying medical conditions compared to healthy children.

In Thailand, increasing evidence suggests that influenza virus plays an important role in childhood respiratory illness. A population-based study in rural Thailand documented that persons hospitalized with influenza pneumonia were more likely to be young children or persons aged ≥65 years old compared to the general Thai population.[10] In this study, the annual incidence of influenza A in children aged <60 months with radiographically-confirmed pneumonia was 90 per 100,000 persons during 2003–2005.[11] However, there are few data on the incidence of influenza among children in urban Bangkok, where almost one fifth of the national population resides.

The Thai Ministry of Public Health currently recommends annual influenza vaccination for certain target groups, including children aged 6 months to <36 months. Each year, approximately three million doses of trivalent inactivated influenza vaccine are provided free of charge by the government to the estimated 11 million high-risk individuals (excluding healthcare workers which are covered by different source of vaccine) on a first-come, first-served basis. Besides this, vaccine is also available for purchase in both public and private sectors. Although recommended, influenza vaccine is not included the routine Expanded Program on Immunization schedule which comprises the required vaccinations for children in Thailand, and is considered an optional vaccine. Finding suggested only 1–2% of children aged 6 months to <36 months were immunized nationwide in recent years.[12] To inform policy decision with respect to influenza disease burden and associated cost in young children and to support the continued inclusion of children as the recommended group for influenza vaccination, we conducted an observational cohort study of Thai children in Bangkok who were enrolled before 36 months of age and followed for up to 24 months to estimate incidences and costs of influenza among healthy children compared to those with underlying medical conditions.

## Methods

### Ethical consideration

The study was approved by the ethics committees of the Queen Sirikit National Institute of Child Health (QSNICH; Bangkok, Thailand) and the Armed Forces Research Institute of Medical Sciences (AFRIMS; Bangkok, Thailand), with the U.S. Centers for Disease Control and Prevention’s Institutional Review Board relied on the QSNICH’s determination. Written parental informed consent was sought for all children.

### Study population

The study population consisted of children receiving healthcare at the QSNICH, the largest children’s tertiary care public hospital in Thailand serving an exclusively pediatric population from birth to 18 years of age. All study participants were residents of the metropolitan area of Bangkok and its vicinity.

### Study design

This study used a prospective cohort design with rolling enrollment of children aged <36 months and aimed to enroll 500 pairs of healthy and high-risk children. Sample size calculation was based on the assumptions that influenza attack rates among healthy and high-risk children were 5% and 10%, respectively, and a type I error of 5% (one-sided hypothesis). With these assumptions, we would need to enroll 381 healthy children and 381 high-risk children to be able to reject the null hypothesis that the attack rates for healthy and high-risk children were equal with probability (power) 0.8. With expected 20% cohort attrition (i.e., 10% per year) and the round-up, 500 pairs of children would be needed. Healthy children were matched by calendar time and age at enrollment with children with underlying medical conditions. Each child whose parent/guardian provided written informed consent was followed up for 24 months (i.e., until up to <60 months of age) with weekly surveillance for acute respiratory illness (ARI) that included collection of respiratory specimens for testing for influenza and respiratory syncytial viruses (RSV). Enrolled children for whom matches could not be identified were allowed to participate in the study throughout the course of the study. Children exited the cohort when 24 months of observation had concluded or upon request of the caregivers.

### Enrollment and eligibility

Enrollment occurred between August 2011 and September 2013. Children were eligible if they were residents of Bangkok metropolitan area and its vicinity and did not plan to move out over the course of the study, <36 months of age, routinely sought care at QSNICH, and were not acutely ill at enrollment. Children who already had a sibling enrolled in the study were excluded. Children were considered “high-risk” if they had ≥1 of the following conditions: prematurity (born at <37 weeks gestation), low birth weight (<2,500 grams), asthma, cardiovascular (excluding hypertension) or congenital heart disease, chronic lung or airway disease, kidney disease, liver disease, neurologic or neuromuscular disease, hemoglobinopathy, metabolic disease including diabetes, Down’s syndrome, immunosuppressive conditions including long-term steroid use, HIV infection, or cancer. Healthy children were defined as those without any of the abovementioned conditions. High-risk children were enrolled when seeking care at specialty clinics, whereas healthy children were enrolled from the well-baby clinic or other outpatient clinics.

At enrollment, caregivers were asked about children’s medical and breastfeeding histories and were given axillary thermometers and instructions on how to take children’s temperatures. Within two months of enrollment, study staff visited children’s homes to record family and household size, request information on household income and assets, ascertain family smoking status, observe characteristics of children’s homes (type of housing, ownership of durable assets, access to sanitation facilities, and source of water), and record childhood immunizations from children’s vaccine books (including influenza vaccination if ≥6 months old). Latitude and longitude of household location were recorded using a global positioning system to determine distance between residence and the QSNICH.

### Active surveillance for acute respiratory illness and collection of cost data

Caregivers were called weekly and asked about ARI symptoms, defined as presence of ≥2 symptoms (documented fever, cough, sore throat or runny nose) with onset during the preceding seven days, among enrolled children. Caregivers were encouraged to call study staff if children developed an ARI between surveillance calls. Three attempts were made to reach caregivers on different days before the caregivers were considered unreachable for that week. Those missing calls for four consecutive weeks were considered lost to follow-up.

For children with ARI, study staff collected information on their highest measured temperature if they had fever and on the presence of ARI in other family members. A 14-day symptom-free period was required between two ARI episodes. Caregivers were encouraged to bring children with ARI to the QSNICH for examination, respiratory specimen collection, and care. Clinicians determined treatment with antivirals based on national treatment guidelines which state empirical treatment should be given to influenza suspect cases and there is no need to wait for laboratory confirmation. If children received medical attention at another facility, medical records were requested, reviewed, and abstracted.

At the post-illness survey, conducted 1–2 weeks following illness onset, study staff collected information from caregivers on children’s daycare or school absentia, symptom duration, and any hospitalizations or costs associated with illness episodes. Caregivers were asked to provide estimates of all direct medical, laboratory, and transportation costs paid out of pocket as well as the indirect costs of reported lost income or time due to care of ill children. Additionally, study staff obtained costs of medical care covered by health insurance from the QSNICH’s financial department. This medical care cost excluded salary of healthcare personnel paid on monthly basis by the Thai government that was not charged to patients or the health insurance scheme.

### Specimen collection and laboratory testing

Children with ARI had combined nasal and throat swabs collected by study nurses for testing for influenza viruses and RSV by real-time reverse transcription polymerase chain reaction (rRT-PCR) at the AFRIMS.[13]

### Influenza vaccination status

Throughout the follow-up period, the influenza vaccination status of children ≥6 months old was periodically updated using the child’s vaccine book or medical records.

### Analysis

Severity of illness was classified as mild if the child required no more than outpatient care and severe if the child required hospitalization. The influenza season was defined as June through May of the following year (e.g., the 2013 influenza season was June 2013-May 2014).[14]

The Chi-Square test was used to compare baseline demographic, socio-economic (i.e., wealth index calculated using Principle Component Analysis method from characteristics of children’s homes as mentioned above), and clinical characteristics between healthy and high-risk children. Population-averaged Poisson regression models [15–17], adjusting for potential confounders (age at ARI, influenza vaccination, recent history of ARI in the household, and influenza season), were fitted for number of events with person-time entered into the models as an offset. Parsimonious models were constructed (i.e., covariates significantly different in bivariate analyses, but not multivariate models, were not included). Adjusted incidence per 1,000 person-years (PY) of events (e.g., ARI, rRT-PCR-confirmed influenza-associated ARI) was calculated from Poisson parameters using least-squares means methods.[18] The 95% Poisson confidence intervals (95% CIs) were calculated using the exact method. The adjusted incidence for healthy and high-risk children, age group at the time of the ARI episodes, and influenza season was compared in Poisson models. All incidence estimates reported were adjusted incidence unless noted otherwise. Log-transformed lengths of illness (i.e., onset to illness resolution based on caregivers’ reports) in healthy and high-risk children with influenza were compared using Student’s t-test.

All direct medical, laboratory and transportation costs and indirect costs of lost income or time due to care of ill children for an ARI episode were summed. Costs paid by caregivers were collected from post-illness interviews and the value of caregiver time was calculated using the human capital approach.[19] Reported healthcare related costs paid for by the health insurance system were checked against the hospital’s database. For salaried or wage-earning caregivers, daily gross pay was multiplied by the number of days that caregivers missed work to calculate lost income. For caregivers who did not work outside the home, time spent caring for ill children was valued at the minimum daily wage of 300 Baht/day (approximately $9) as established by the Thai government.[20–22] ARI-associated costs that incurred before 2015 were adjusted to the 2015 value using an inflation rate of 3%.[20, 21] Costs were converted using exchange rate of 34.24 Baht to $1 [23] and reported in U.S. dollars in absolute terms and in relation to the median monthly household income of children in the cohort (reported as range of $584-$876/month). The median cost interquartile range of ARI were calculated. Because high-risk children were significantly more likely to live further away and incurred higher travel expense than healthy children, differences in the costs between the two groups were compared by quantile regression adjusted for distance from residence to the QSNICH.[24] All data analyses were conducted using Stata software version 12 (StataCorp LLC, College Station, Texas, USA).

## Results

### Characteristics of study population

During August 2011 and September 2015, 1,149 children were enrolled (649 healthy and 500 high-risk children; **Table 1**). Upon review of medical records, 4 healthy children were reassigned to high-risk group due to the misunderstanding of disease definitions and 14 high-risk children were reassigned to healthy group, resulting in the analytic dataset having 659 healthy and 490 high-risk children. Of these, 621 children (54%) were enrolled at <12 months old and 624 (54%) were males. Healthy children were statistically significantly more likely than high-risk children to have ever been breastfed, live in households with higher monthly income, have primary caregivers with more years of education, live closer to QSNICH, live in an apartment complex, have electricity and air condition in the house, and pay for the illness out of pocket. For children who were eligible for influenza vaccination at the beginning of each season (i.e., ≥6 months of age), the coverage ranged from 3% to 29% in 2011 through 2015 (Table 1). The coverage was comparable in healthy and high-risk children, except in 2011, 2012, and 2015. Of all children, 287 (25%) had one underlying medical condition, 110 (10%) had two, 61 (5%) had three, and 32 (3%) had >3 conditions **(S1 Table)**. Among those with a single condition, the most common conditions were prematurity (170; 15%), congenital heart disease (43; 4%), and respiratory disease (22; 2%). Nine hundred and one children (78%) completed the 24-month follow-up successfully (522 [80%] healthy vs. 379 [76%] high-risk children; p-value = 0.10).

**Table 1:** Socio-economic and baseline characteristics of children enrolled in a pediatric respiratory infection cohort in Thailand.

	Healthy	High-risk	All	P-value
	(total = 659)	(total = 490)	(total = 1,149)	
	N (%)	N (%)	N (%)	
Age at enrollment (months)				0.41
*0–11*	345 (52)	276 (56)	621 (54)	
*12–23*	184 (28)	125 (26)	309 (27)	
*24–35*	130 (20)	89 (18)	219 (19)	
Male	344 (52)	280 (57)	624 (54)	0.1
Ever breastfed at enrollment	611 (93)	430 (89)	1,041 (91)	<0.01
Enrolled child attend daycare or school at enrollment	82 (13)	45 (9)	127 (11)	0.08
Daycare attendance at enrollment by age^γ^				0.12
*<6 months*	10 (2)	11 (2)	21 (2)	
*6–11 months*	17 (3)	7 (1)	24 (2)	
*12–17 months*	9 (1)	7 (1)	16 (1)	
*18–23 months*	6 (1)	6 (1)	12 (1)	
*24–29 months*	18 (3)	7 (1)	25 (2)	
*30–35 months*	22 (3)	7 (1)	29 (2)	
Monthly household income in Thai Baht				<0.01
*1–9*,*999 ($292)*	54 (8)	70 (14)	124 (11)	
*10*,*000–19*,*999 ($294-$584)*	180 (27)	166 (34)	346 (30)	
*20*,*000–29*,*999 ($284-$876)*	142 (22)	101 (21)	243 (21)	
*30*,*000–39*,*999 ($876-$1*,*168)*	108 (16)	61 (12)	169 (15)	
*≥40*,*000 (≥$1*,*169)*	163 (25)	83 (17)	236 (21)	
*Not answered*	12 (2)	9 (2)	21 (2)	
Primary caregiver’s highest education				<0.01
*No schooling*	13 (2)	11 (2)	24 (2)	
*Primary school*	102 (15)	120 (24)	222 (19)	
*Secondary school*	257 (39)	197 (40)	454 (39)	
*Vocational school*	108 (16)	67 (14)	175 (15)	
*University*	164 (25)	86 (18)	250 (22)	
*Missing*	15 (2)	9 (2)	24 (2)	
Distance from home to QSNICH (km)^γ^				<0.01
*0–10*	354 (55)	175 (36)	529 (47)	
*>10–20*	186 (28)	137 (28)	323 (29)	
*>20–30*	78 (12)	108 (22)	186 (16)	
*>30–40*	23 (3)	45 (9)	68 (6)	
*>40*	6 (1)	17 (4)	23 (2)	
Wealth index^γ^				0.08
*1*^*st*^ *quintile*	121 (19)	121 (25)	242 (22)	
*2*^*nd*^ *quintile*	117 (18)	91 (19)	208 (18)	
*3*^*rd*^ *quintile*	140 (22)	87 (18)	227 (20)	
*4*^*th*^ *quintile*	135 (21)	93 (19)	228 (20)	
*5*^*th*^ *quintile*	133 (21)	87 (18)	220 (20)	
Rule about smoking inside the house^γ^				0.91
*Allowed inside the house*	33 (5)	23 (5)	56 (5)	
*Generally not allowed*, *but there were exceptions*	33 (5)	27 (6)	60 (5)	
*Never allowed inside the house*	581 (90)	431 (89)	581 (90)	
Coverage of ARI related expense^€^				<0.01
*Out of pocket*	1,485 (81)	736 (57)	2,221 (72)	
*Civil servant scheme*	183 (10)	72 (6)	255 (8)	
*Universal coverage scheme*	30 (2)	193. (15)	223 (7)	
*Borrowed money*	4 (<1)	1 (<1)	5 (<1)	
*Private health insurance*	6 (<1)	3 (<1)	9 (<1)	
*Combined methods*	95 (5)	73 (6)	168 (5)	
*Others*^*μ*^	25 (1)	202 (16)	227 (7)	
Influenza vaccination status^£^				
*Vaccinated in 2010 season*	10 (14)	7 (14)	17 (14)	1
*Unvaccinated in 2010 season*	62 (86)	42 (86)	104 (86)	
*Vaccinated in 2011 season*	51 (25)	47 (36)	98 (29)	0.03
*Unvaccinated in 2011 season*	156 (75)	85 (64)	241 (71)	
*Vaccinated in 2012 season*	137 (32)	67 (23)	204 (28)	<0.01
*Unvaccinated in 2012 season*	294 (68)	228 (77)	522 (72)	
*Vaccinated in 2013 season*	153 (26)	103 (23)	256 (25)	0.27
*Unvaccinated in 2013 season*	444 (74)	334 (77)	778 (75)	
*Vaccinated in 2014 season*	72 (11)	59 (12)	131 (11)	0.6
*Unvaccinated in 2014 season*	587 (89)	431 (88)	1,018 (89)	
*Vaccinated in 2015 season*	13 (2)	19 (4)	32 (3)	0.04
*Unvaccinated in 2015 season*	646 (98)	471 (96)	1,117 (97)	

QSNICH, Queen Sirikit National Institute of Child Health; ARI, acute respiratory illness

^γ^Among 1,129 children whose household visits were conducted; wealth index was created among 1,125 children with complete data using the following variables: housing characteristics, ownership of durable assets, access to sanitation facilities, and source of water

^€^From 3,108 ARIs

^μ^Company welfare, health scheme for handicaps, or no expense (used existing medicines or sought care at health centers

^£^Among children who were ≥6 months of age at the beginning of the season: 121 children for 2010 season (72 healthy and 49 high-risk), 339 for 2011 season (207 healthy and 132 high-risk), 726 for 2012 season (431 healthy and 295 high-risk), 1,034 children for 2013 season (597 healthy and 437 high-risk), and 1,149 for 2014 and 2015 seasons (659 healthy and 490 high-risk)

### ARI episodes and detection of influenza viruses and RSV

**Fig 1:**
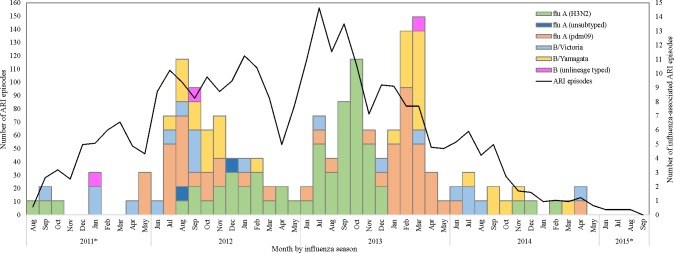
Number of acute respiratory illnesses (ARI) and influenza-associated ARI among children enrolled in a pediatric respiratory infection cohort in Thailand. ARI: acute respiratory illness. *partial season.

We identified 3,108 ARI cases in 861 children **(Fig 1)**; 2,787 (90%) of which sought care at QSNICH. ARI cases occurred year-round, but were most frequent in July-October (1,472 episodes) and December-March (1,068 episodes). We collected and tested 2,833 (91.2%) combined nasal and throat specimens. Influenza viruses or RSV were identified in 536 (18.9%) samples: 156 (5.5%) influenza, 372 (13.1%) RSV, and 8 (0.3%) co-infections. Among 164 influenza virus-positive specimens, 111 (68%) were influenza A and 53 (32%) were influenza B **(Fig 1)**. The influenza A viruses identified were subtypes H3N2 (59/111, 53%) and H1N1pdm09 (50/111, 45%), and two of 111 (2%) had too low viral load to be subtyped. Of the 53 B viruses, we identified 31 (58%) as the Yamagata lineage and 19 (36%) as the Victoria lineage while the lineage of the remaining 3 (6%) could not be determined. Influenza B virus predominated in the 2011, 2012, and 2014 seasons while influenza A subtype H3N2 virus predominated 2013 season.

### Incidence of influenza-associated ARI

The enrolled children contributed 1,980 person-years (PY) during the 24-month follow-up, resulting in a crude ARI incidence of 1,569/1,000 PY (95% CI: 1,515–1,626). The adjusted ARI incidence was similar between healthy and high-risk children (1,205/1,000 PY; 95% CI: 1,113–1,305 vs. 1,273/1,000 PY; 95% CI: 1,165–1,390).

The crude and adjusted incidence of influenza-associated ARI is shown in S2 Table. The incidence of influenza-associated ARI adjusted for age at ARI, influenza vaccination status, recent history of ARI in the household, and influenza season was 41/1,000 PY (95% CI: 33–52) and was statistically higher in healthy than high-risk children (48/1,000 PY; 95% CI: 38–62 vs. 33/1,000 PY; 95% CI: 24–46). The incidence rate ratio [IRR] was 1.45 (95% CI: 1.01–2.10).

The incidence of mild influenza-associated ARI adjusted for age at ARI, influenza vaccination status, recent history of ARI in the household, and influenza season was 37/1,000 PY (95% CI: 29–47) and was statistically higher in healthy than high-risk children (45/1,000 PY; 95% CI: 34–58 vs. 27/1,000 PY; 95% CI: 18–38). The incidence rate ratio was 1.67 (95% CI: 1.13–2.48). The incidence of mild influenza-associated ARI adjusted for influenza vaccination status, recent history of ARI in the household, and influenza season was lowest in infants aged <12 months and highest in those aged 36–47 months **(Table 2A)**. In both healthy and high-risk children, the incidence of mild influenza-associated ARI adjusted for age at ARI, influenza vaccination status, and recent history of ARI in the household was highest in 2013 and lowest in 2012 **(Table 2B)**.

**Table 2:** Incidence per 1,000 person-years of mild and severe influenza-associated acute respiratory illness in children enrolled in a pediatric respiratory infection cohort in Thailand^γ^.

	Healthy		High-risk		All	
	Influenza cases	Adjusted incidence (95% confidence interval)	Influenza cases	Adjusted incidence (95% confidence interval)	Influenza cases	Adjusted incidence (95% confidence interval)
***A*. *Incidence by age***						
**Mild or severe**						
0–11 months	13	32 (18–59)	5	23 (12–42)	18	28 (16–49)
12–23 months	43	51 (36–71)	21	35 (24–52)	64	44 (32–61)
24–35 months	38	49 (34–70)	14	34 (22–51)	52	42 (30–59)
36–47 months	18	57 (36–91)	6	40 (24–66)	24	51 (32–79)
48–59 months	4	45 (17–123)	2	31 (11–87)	6	40 (15–107)
**Mild**						
0–11 months	12	31 (17–57)	5	19 (10–36)	17	25 (14–46)
12–23 months	40	47 (33–67)	17	28 (18–43)	57	39 (28–55)
24–35 months	35	44 (30–64)	11	27 (17–41)	46	36 (25–52)
36–47 months	16	52 (32–84)	5	31 (18–54)	21	44 (27–70)
48–59 months	4	45 (16–122)	1	27 (10–76)	5	38 (14–102)
**Severe**						
0–11 months	1	1 (<1–12)	0	3 (<1–27)	1	2 (<1–17)
12–23 months	3	3 (1–11)	4	7 (2–22)	7	5 (2–13)
24–35 months	3	3 (1–13)	3	9 (3–27)	6	6 (2–16)
36–47 months	2	4 (1–20)	1	10 (2–44)	3	6 (1–26)
48–59 months	0	0	1	0	1	0
***B*. *Incidence by season***						
**Mild or severe**						
2012	40	44 (30–63)	18	30 (19–46)	58	38 (27–54)
2013	50	67 (50–91)	29	46 (32–66)	79	58 (44–76)
2014	15	39 (22–68)	1	26 (14–48)	16	34 (19–59)
**Mild**						
2012	35	39 (27–58)	15	23 (15–37)	50	33 (23–47)
2013	49	62 (45–85)	23	37 (25–55)	72	51 (38–69)
2014	14	40 (23–71)	1	24 (13–44)	15	33 (19–58)
**Severe**						
2012	5	*	3	*	8	*
2013	1	*	6	*	7	*
2014	1	*	0	*	1	*

Incidence was adjusted for influenza vaccination status, recent history of ARI in the household, and influenza season in (A) and for age at acute respiratory illness, influenza vaccination status, and recent history of ARI in the household in (B).

^γ^Mild, cases treated in outpatient department; severe, cases treated in inpatient department

*model not converged

The adjusted incidence of severe influenza-associated ARI was 4/1,000 PY (95% CI: 2–9). The incidence of severe influenza-associated ARI was 3/1,000 PY; 95% CI: 1–8 among healthy children and 7/1,000 PY; 95% CI: 3–16 among high-risk children. The incidence rate ratio was 0.40 (95% CI: 0.11–1.38). The adjusted incidence of severe influenza-associated ARI was lowest in children aged 48–59 months and highest in those aged 36–47 months **(Table 2A)**. We could not reliably calculate the adjusted incidence of severe influenza-associated ARI by risk group and influenza season due to the low number of influenza cases.

### Length of ARI and influenza-associated ARI

Among 3,092 ARI episodes that resolved by the time of the post-illness interviews (99% of ARIs identified), the median length of illness was 8 days (IQR: 8–11) among all children with no difference in illness duration among healthy versus high-risk children (8 days; IQR: 8–11 vs. 8 days; IQR: 7–12; p-value for difference in medians = 0.06). The length of mild influenza-associated ARI was 9 days (IQR 8–12) among all children, with no difference in illness duration among healthy versus high-risk children (9 days; IQR: 7–12 vs. 10 days; IQR: 8–12; p-value for difference in medians = 0.34). The length of severe influenza-associated ARI was 10 days (IQR: 9–15) among all children, with no difference in illness duration among healthy versus high-risk children (9 days; IQR: 7–12, vs. 11 days; IQR: 9–13; p-value for difference in medians = 0.84).

### Cost of influenza-associated ARI

**Table 3** shows the median cost per ARI episode adjusted for distance from residence to the study site. In general, the adjusted median costs were similar in healthy and high-risk children. The adjusted median costs for mild influenza-associated ARI was approximately 2–4% of the monthly household income of the children while that of severe influenza-associated ARI ranged from 28% to 42% of monthly household income. Among mild influenza cases, 37% of the total adjusted cost per episode was attributable to healthcare related direct cost (i.e., medicine, laboratory testing, etc.), 36% to non-healthcare related direct cost i.e., travel cost, and 27% to indirect cost (22% opportunity cost and 5% reported income loss). Among severe influenza cases, 82% of the total adjusted cost per episode was attributable to healthcare related direct cost, 7% to non-healthcare related direct cost, and 11% to indirect cost (9% opportunity cost and 2% reported income loss).

**Table 3:** Median cost per acute respiratory illness episode adjusted for distance from residence to the study site in children enrolled in a pediatric respiratory infection cohort in Thailand.

	Adjusted median cost per episode (interquartile range)^γ^						Difference in adjusted medians* (95% confidence interval)
	Number	Healthy	Number	High-risk	Number	All	
ARI episode	1828	20 (18–24)	1280	23 (22–25)	3108	20 (18–23)	1 (-0.5 to 3)
Non-influenza associated ARI	1712	20 (18–24)	1232	23 (21–25)	2944	21 (19–24)	2 (-0.4 to 3)
Influenza associated ARI	116	23 (20–27)	48	23 (22–25)	164	25 (23–28)	4 (-5 to 14)
Mild influenza-associated ARI	107	22 (19–25)	39	25 (23–26)	146	22 (21–24)	-1 (-9 to 6)
Severe influenza-associated ARI	9	232 (211–243)	9	318 (159–607)	18	246 (235–258)	110 (-352 to 571)

^γ^Median costs were adjusted for distance from residence to the study site and reported in US dollar.

*Differences in the adjusted median costs between the two groups were compared by quantile regression.

ARI: acute respiratory illness

## Discussions

In this study spanning three full influenza seasons (2012–2014), healthy children had a significantly higher incidence of mild influenza-associated ARI than children with high-risk conditions. The length of illness and cost of influenza-associated ARI were similar in healthy and high-risk children. Costs per episode of severe influenza-associated ARI, which may be borne either by the families or the healthcare system, were substantial.

The overall incidence of mild influenza-associated ARI among healthy children in this study (45/1,000 PY) was comparable to the most recent global estimate of influenza incidence among children aged <60 months in developed countries (55/1,000 PY) [25], possibly reflecting the high influenza vaccination coverage among cohort children. However, our overall incidence estimate was lower than estimates reported in some single country studies conducted among children of the same age group.[26, 27] For example, a study among community-dwelling children aged 6–59 months in Wisconsin, USA in 2006–2010, reported an estimated incidence of influenza-associated outpatient visits of 77/1,000 PY.[26] Another study conducted in Suzhou, China reported an influenza virus infection incidence among children aged <60 months ranging from 146 to 214/1,000 PY for the 2011 to 2014 seasons.[27] In studies conducted among children aged <60 months in Bangladesh (2009–2011) and India (2010–2012), higher incidence of severe influenza virus infection was reported.[28, 29] The differences in incidences in these studies may be attributable to differences in study design, geographic and climate pattern, source population, influenza vaccination coverage, and severity of influenza seasons during which the studies were conducted.

In this study, the higher incidence of mild influenza-associated ARI in healthy compared to high-risk children may seem counter-intuitive, but healthy children might have been allowed to come into greater contact with contagious ARI cases than high-risk children. There was a trend towards healthy children in our study being more likely to attend daycare than high-risk children. Healthy children in our study were also more likely to live in buildings with other households potentially allowing for more comingling with other children and increasing the risk of exposure to respiratory infections. Our study did not identify a statistically significant difference in the incidence of severe ARI between healthy and high-risk children but this study may not have been powered to detect meaningful difference in severe influenza incidence between the two groups. Additionally, for the 2012, 2013, and 2014 seasons with complete follow-up data, we did not identify a statistically significant difference in the estimated incidence across the three seasons.

Our study showed that the cost of treating an episode of severe influenza-associated ARI could be as high as >$200/episode. While this cost is lower than figures reported from studies conducted in high-income countries [30, 31], it is higher than that reported in other middle income countries such as Bangladesh and India (both of which reported total cost of less than $100/episode).[32, 33] We found that cost of treating an episode of severe influenza-associated ARI is about two fifths of the median monthly household income of children in the cohort. This sizable cost is either borne by the parents/caregivers themselves or the healthcare system. This finding supports the benefit of annual influenza vaccination in reducing the burden of influenza and associated cost.

Our study has several strengths. It was conducted in a defined cohort with nearly 100% of ARI episodes managed at the QSNICH, reducing potential for missed influenza cases. Children with underlying medical conditions were oversampled, enabling us to estimate incidence of influenza in this sub-population. Further, we used rRT-PCR, an assay that is maximally sensitive and specific for influenza viruses, to ascertain influenza virus infection status, thus minimizing misclassification bias.

However, several limitations should be considered when interpreting our findings. First, all children with ARI were encouraged to visit the study hospital including those with mild symptoms who might not require a hospital visit and about 10% of ARI cases received empirical treatment using Oseltamivir (of which 64% were tested positive based on rapid influenza diagnostic testing) [34], thereby lessening disease severity. Therefore, the associated costs may be imprecise. Second, influenza vaccination coverage was higher among cohort children than estimates among the general Thai pediatric population (1–2%), possibly because the QSNICH is an academic pediatric facility that emphasizes influenza vaccination for children seeking care at the facility. The higher influenza vaccination coverage may have resulted in a lower incidence of influenza among cohort children compared to the general population. Third, the study was not powered to measure the incidences of influenza outcomes by type of underlying medical condition. It was also not powered to measure the incidences of severe influenza outcomes.

Data are needed to guide prioritization of target groups for seasonal influenza vaccination in less wealthy countries where influenza vaccine supplies may not always be adequate to cover all recommended target groups. In Thailand, influenza vaccination is recommended for children aged 6 months to <36 months, the vaccine is given infrequently to children in this age group who do not have underlying conditions. Our finding that healthy children have a higher incidence of mild influenza-associated ARI than high-risk children could be used to generate cost-effectiveness data and to estimate the marginal value of increasing influenza vaccine coverage among children aged 6 months to <36 months. Additionally, the finding that children >36 months of age have similar influenza incidence as younger children suggests the potential utility of evaluating the marginal benefit of expanding influenza vaccine use to older children.

## Supporting information

S1 Table Number and types of underlying medical conditions among high-risk children in the cohort. (DOCX)

S2 Table Crude and adjusted incidence of influenza-associated acute respiratory illness among children enrolled in a pediatric respiratory infection cohort in Thailand. Incidence was adjusted for age at ARI, influenza vaccination status, recent history of ARI in the household, and influenza season. ARI: Acute respiratory illness.
(DOCX)

S1 File Dataset. (XLSX)
