# Supplementary material for: Comparison of incidence and cost of influenza between healthy and high-risk children <60 months old in Thailand, 2011-2015
Source: PLoS One. 2018 May 17;13(5):e0197207. doi: 10.1371/journal.pone.0197207 (PMC5957403; doi:10.1371/journal.pone.0197207)
Supplement: S1 Table — (DOCX) [file pone.0197207.s001.docx]

**S1 Table 1. Number and types of underlying medical conditions among high-risk children in the cohort.**

| Underlying medical condition | Frequency (% of total children in the cohort) |
| --- | --- |
| One condition | 287 (25.0) |
| Born at <37 gestational weeks or birth weight <2,500 grams | 170 (14.8) |
| Congenital heart or circulatory disease (excluding hypertension) | 43 (3.7) |
| Respiratory disease (e.g., asthma, chronic lung or airway disease, abnormality of the upper airway) | 24 (2.1) |
| Developmental delay (e.g., Down’s syndrome) | 17 (1.5) |
| Neurologic/neuromuscular disorder (including muscular dystrophy, cerebral palsy) | 10 (0.9) |
| Hemoglobinopathy including thalassemia | 6 (0.5) |
| Metabolic disease (including diabetes) | 6 (0.5) |
| Kidney disease | 5 (0.4) |
| HIV infection | 3 (0.3) |
| Cancer | 2 (0.2) |
| Liver | 1 (0.1) |
| Two conditions | 110 (9.2) |
| Three conditions | 61 (5.3) |
| Four conditions | 18 (1.6) |
| More than four conditions | 14 (1.2) |
