# Supplementary material for: Comparison of incidence and cost of influenza between healthy and high-risk children <60 months old in Thailand, 2011-2015
Source: PLoS One. 2018 May 17;13(5):e0197207. doi: 10.1371/journal.pone.0197207 (PMC5957403; doi:10.1371/journal.pone.0197207)
Supplement: S2 Table — Incidence was adjusted for age at ARI, influenza vaccination status, recent history of ARI in the household, and influenza season. ARI: Acute respiratory illness. (DOCX) [file pone.0197207.s002.docx]

**S2 Table. Crude and adjusted incidence of influenza-associated acute respiratory illness among children enrolled in a pediatric respiratory infection cohort in Thailand.**

| Setting | Crude rate per 1,000 person-years | | Adjusted rate per 1,000 person-years | |
| --- | --- | --- | --- | --- |
|  | Estimate (95% confidence interval) | Incidence rate ratio between healthy and high-risk children | Estimate (95% confidence interval) | Incidence rate ratio between healthy and high-risk children |
| **Outpatient and inpatient** |  | | | |
| Influenza associated ARI in all children | 83 (71-97) |  | 41 (33-52) |  |
| Influenza associated ARI among healthy children | 101 (85-121) | 1.77 (1.27-2.48) | 48 (38-62) | 1.45 (1.01-2.10) |
| Influenza associated ARI among high-risk children | 57 (43-76) |  | 33 (24-46) |  |
| **Outpatient** |  | | | |
| Influenza associated ARI in all children | 73 (63-86) |  | 37 (29-47) |  |
| Influenza associated ARI among healthy children | 93 (77-112) | 2.01 (1.40-2.89) | 45 (34-58) | 1.67 (1.13-2.48) |
| Influenza associated ARI among high-risk children | 46 (34-63) |  | 27 (18-38) |  |
| **Inpatient** |  | | | |
| Influenza associated ARI for all children | 9 (6-15) |  | 4 (2-9) |  |
| Influenza associated ARI among healthy children | 8 (4-16) | 0.74 (0.28-1.91) | 3 (1-8) | 0.40 (0.11-1.38) |
| Influenza associated ARI among high-risk children | 11 (5-21) |  | 7 (3-16) |  |
